# Antioxidant, Antimicrobial Properties and Phenolics of Different Solvent Extracts from Bark, Leaves and Seeds of *Pongamia pinnata *(L.) Pierre

**DOI:** 10.3390/molecules17043917

**Published:** 2012-03-30

**Authors:** Zahid Iqbal Sajid, Farooq Anwar, Ghulam Shabir, Ghulam Rasul, Khalid M. Alkharfy, Anwarul-Hassan Gilani

**Affiliations:** 1 Department of Chemistry and Biochemistry, University of Agriculture, Faisalabad 38040, Pakistan; Email: zi_sajid@yahoo.com (Z.I.S.); gshabirnibge@yahoo.com (G.S.); 2 Agriculture Biotechnology Division, National Institute for Biotechnology and Genetic Engineering (NIBGE), P.O. Box 577, Jhang Road, Faisalabad, Pakistan; Email: ghulamrasul_pk@yahoo.co.uk; 3 Department of Chemistry, University of Sargodha, Sargodha 40100, Pakistan; 4 Environmental Biotechnology Division, National Institute for Biotechnology and Genetic Engineering (NIBGE), P.O. Box 577, Jhang Road, Faisalabad, Pakistan; 5 Department of Clinical Pharmacy, College of Pharmacy, King Saud University, Riyadh 11451, Saudi Arabia; Email: alkharfy@ksu.edu.sa; 6 Natural Products Research Division, Department of Biological and Biomedical Sciences, Aga Khan University Medical College, Karachi 74800, Pakistan

**Keywords:** *P. pinnata*, solvent extracts, antioxidants, antimicrobial, HPLC, phenolic acids

## Abstract

This study appraises the antioxidant and antimicrobial attributes of various solvent extracts (absolute methanol, aqueous methanol, absolute ethanol, aqueous ethanol, absolute acetone, aqueous acetone, and deionized water) from bark, leaves and seeds of *Pongamia pinnata *(L.) Pierre. Maximum extraction yield of antioxidant components from bark (16.31%), leaves (11.42%) and seeds (21.51%) of *P. pinnata* was obtained using aqueous methanol (20:80). Of the extracts tested, the bark extract, obtained with aqueous methanol, exhibited greater levels of total phenolics [6.94 g GAE/100 g dry weight (DW)], total flavonoids (3.44 g CE/100 g DW), inhibition of linoleic acid peroxidation (69.23%) and DPPH radical scavenging activity (IC_50_ value, 3.21 μg/mL), followed by leaves and seeds extracts. Bark extract tested against a set of bacterial and fungal strains also revealed the strongest antimicrobial activity with the largest inhibition zone and lowest minimum inhibitory concentration (MIC). HPLC analysis of aqueous methanol extracts from bark, leaves and seeds indicated the presence of protocatechuic, ellagic, ferulic, gallic, gentisic, 4-hydroxybenzoic and 4-hydroxycinnamic acids in bark (1.50–6.70 mg/100 g DW); sorbic, ferulic, gallic, salicylic and *p*-coumaric acids in leaves (1.18–4.71 mg/100 g DW); vanillic, gallic and tannic acids in seeds (0.52–0.65 mg/100 g DW) as the main phenolic acids. The present investigation concludes that the tested parts of *P. pinnata*, in particular the bark, have strong potential for the isolation of antioxidant and antimicrobial agents for functional food and pharmaceutical uses.

## 1. Introduction

Currently there is much interest in the uses of plant-based natural antioxidants, especially the phenolic acids and flavonoids, because of their functional food and nutraceutical potential [1]. Such natural substances possess anticarcinogenic activity and offer diverse health-promoting effects due to their antioxidant and radical scavenging properties [1,2]. In this context, a huge number of medicinal plants are known to produce such bioactives with potential antioxidant and antimicrobial activities [3,4]. The phenolic acids, which can inhibit pathogens growth and have little toxicity to host cells are also promising candidates for developing new antimicrobial drugs. Consequently, there is growing interest in developing many plant-derived drugs with multiple biological functions to use for the treatment of various infectious diseases [5,6]. 

*Pongamia pinnata *(L.) Pierre [Synonyms: *Derris indica* (Lam.) Bennett, *Derris pinnata *Lour, Millettia novo-guineensis Kane and Hat, *Pongamia glabra* Vent, *Cytisus pinnatus *L, *Pongamia pinnata* Merr.] belonging to the family Fabaceae (Papilionaceae), is widely distributed in tropical Asia, Australia, Polynesia and Philippine Islands. In Pakistan, this plant is locally known as “Sukh Chain” and is cultivated in all the four provinces of the country. Traditionally, different parts of *P. pinnata* such as bark, leaves, seeds, roots, flowers and stem have been utilized in the native medicine systems of different civilizations [7]. The flowers of this plant have been found to possess anti-hyperglycemic and anti-lipid peroxidation properties [8]. Its bark is used in piles; leaves are effective as a medicated bath and in rheumatic pains while the seeds are used in hypertension, bronchitis, whooping cough, skin diseases and rheumatic arthritis [9,10,11]. Roots are used for cleaning gums, teeth, and ulcers and also effective in gonorrhea [12,13]. Flavones, isoflavones, chalcones, furanoflavonoids and pyranoflavonoids have been reported as the main phenolic constituents from various parts of the investigated plant [10,14,15,16,17]. A furanoflavone, karanjin, isolated from the seeds of this plant possesses insecticidal and antibacterial properties. Alcoholic extracts of* P. pinnata *seed oil showed activity against both Gram positive and Gram negative bacteria. The oil has been applied in scabies, herpes, leucoderma and other cutaneous diseases. Internally, it has sometimes been used as a stomachic and cholagogue in case of dyspepsia with sluggish liver [10].

Although several reports have revealed the medicinal uses of this valuable plant, however to the best of our knowledge, no detailed work has been conducted so far on the antioxidant and antimicrobial properties of bark, leaves and seeds of *P. pinnata* indigenous to Pakistan. As a part of our systematic studies on the investigation of antioxidant and biological attributes of local medicinal plants [18,19], the present study was undertaken with the main objective to screen different parts of this potential plant for antioxidant and antimicrobial attributes and to determine their individual phenolic acid profiles using HPLC.

## 2. Results and Discussion

### 2.1. Extract Yields

The extract yields of antioxidant components from bark, leaves and seeds of *P. pinnata* by different extraction solvents are shown in Table 1. The extract yields from bark, leaves and seeds varied from 1.92–16.31, 1.50–11.42 and 5.44–21.51 g/100 g of dry weight (DW), respectively, showing significant differences among the parts tested. Within the different parts and extraction solvents, aqueous methanol seeds extract showed the maximum yield followed by bark and leaves extracts. The extracting ability of different solvents for the parts tested followed the order: aqueous methanol > aqueous ethanol > absolute ethanol > absolute methanol > deionized water > aqueous acetone > absolute acetone. These trends are supported by the investigation of Siddhuraju and Becker [20], who revealed that aqueous methanol and aqueous ethanol are effective solvents to extract antioxidant compounds from plant material [20]. Significant (*p *< 0.05) differences of extract yield among different solvents and plant parts might be attributed to the varied polarity of solvents as well as the availability of different extractable components in each part of plant.

### 2.2. Total Phenolics Content (TPC)

The TPC, recovered from bark, leaves and seeds extracts of* P. pinnata*, ranged from 1.19–6.94, 0.89–3.83, and 0.06–0.71 g GAE/100 g DW, respectively (Table 1). Among different solvent tested, aqueous methanol showed excellent efficacy towards extraction of maximum TPC from bark (6.94 g GAE/100 g DW) followed by leaves (3.83 g GAE/100 g DW) and seeds (0.71 g GAE/100 g DW). The TPC of selected plant parts varied significantly (*p* < 0.05) among different solvent extracts. The TPC of the bark and leaves were higher than those investigated in a previous study for methanolic extract of* P. pinnata *stem (1.02 g/100 g DW) and leaf (1.22 g/100 g DW), however, the present values for seeds were lower than that reported previously (0.96 g/100g DW) [21]. The present amount of total phenolics in the leaves were also higher than those reported previously for methanolic extract of leaves (0.86 g GAE /100g) of *P. pinnata* [22,23]*.* Variation in the phenolic contents of various solvent extracts might be attributed to the polarities of different solvents as well as the chemical nature of the endogenous extractable compounds [24]. 

**Table 1:** Yields and antioxidant activity of different solvent extracts from bark, leaves, and seeds of *P. pinnata*.

Parameters	Solvent extracts						
*Bark*	Absoluteethanol	Aqueous ethanol	Absolutemethanol	Aqueous methanol	Absoluteacetone	Aqueous acetone	Deionizedwater
Yield (%)	12.01 ± 0.36 ^b^	12.51 ± 0.46 ^ab^	10.02 ± 0.35 ^ab^	16.31 ± 0.45 ^a^	1.92 ± 0.04 ^e^	4.91 ± 0.15 ^d^	9.70 ± 0.23 ^c^
Total phenolic content (g GAE/100 g DW)	3.21 ± 0.02 ^d^	4.22 ± 0.04 ^c^	5.11 ± 0.03 ^b^	6.94 ± 0.04 ^a^	1.19 ± 0.02 ^e^	2.21 ± 0.03 ^c^	1.21 ± 0.01 ^e^
Total flavonoid content (g CE/100 g DW)	1.26 ± 0.01 ^bc^	2.28 ± 0.01 ^b^	2.34 ± 0.02 ^ab^	3.44 ± 0.04 ^a^	0.92 ± 0.01 ^c^	1.06 ± 0.01 ^c^	0.85 ± 0.01 ^c^
DPPH, IC_50_ (μg/mL)	7.13 ± 0.36 ^ab^	6.18 ± 0.28 ^b^	5.14 ± 0.42 ^bc^	3.21 ± 0.16 ^c^	10.42 ± 0.30 ^a^	8.58 ± 0.37 ^ab^	10.01 ± 0.25 ^a^
Inhibition of linoleic acid peroxidation (%)	37.37 ± 1.70 ^bc^	44.52 ± 3.51 ^bc^	48.23 ± 2.33 ^b^	69.23 ± 1.62 ^a^	28.47 ± 1.81 ^c^	32.12 ± 2.54 ^c^	20.51 ± 1.42 ^d^
Reducing power for 10 mg/mL extract conc.	1.25 ± 0.04 ^b^	1.41 ± 0.04 ^ab^	1.58 ± 0.03 ^a^	1.73 ± 0.05 ^a^	0.79 ± 0.03 ^c^	0.93 ± 0.06 ^cd^	0.55 ± 0.04 ^d^
***Leaves***							
Yield (%)	9.81 ± 0.51 ^b^	10.12 ± 0.81 ^a^	8.84 ± 0.32 ^b^	11.42 ± 0.74 ^a^	1.50 ± 0.02 ^d^	4.72 ± 0.19 ^c^	8.63 ± 0.52 ^b^
Total phenolic content (g GAE/100 g DW)	2.02 ± 0.05 ^ab^	2.81 ± 0.08 ^b^	2.40 ± 0.07 ^b^	3.83 ± 0.12 ^a^	1.01 ± 0.02 ^bc^	1.21 ± 0.05 ^bc^	0.89 ± 0.04 ^c^
Total flavonoid content (g CE/100 g DW)	0.26 ± 0.01 ^ab^	0.34 ± 0.05 ^b^	0.38 ± 0.06 ^b^	0.61 ± 0.05 ^a^	0.10 ± 0.01 ^c^	0.22 ± 0.05 ^b^	0.18 ± 0.01 ^c^
DPPH, IC_50_ (μg/mL)	8.16 ± 0.16 ^bc^	7.83 ± 0.25 ^c^	6.0 ± 0.31 ^c^	4.42 ± 0.03 ^d^	12.12 ± 1.3 ^ab^	10.03 ± 0.15 ^b^	16.46 ± 0.25 ^a^
Inhibition of linoleic acid peroxidation (%)	32.71 ± 1.72 ^bc^	36.22 ± 1.21 ^b^	42.14 ± 1.52 ^ab^	50.65 ± 2.24 ^a^	21.44 ± 2.2 ^cd^	25.43 ± 1.20 ^c^	16.85 ± 2.0 ^d^
Reducing power for 10 mg/mL extract conc.	1.18 ± 0.04 ^b^	1.36 ± 0.04 ^ab^	1.52 ± 0.03 ^ab^	1.64 ± 0.04 ^a^	0.62 ± 0.03 ^c^	0.79 ± 0.05 ^cd^	0.43 ± 0.02 ^d^
***Seeds***							
Yield (%)	18.02 ± 2.1 ^ab^	19.70 ± 1.8 ^ab^	14.61 ± 0.41 ^b^	21.51 ± 1.6 ^a^	5.44 ± 0.35 ^c^	12.6 ± 0.50 ^ab^	14.40 ± 0.69 ^b^
Total phenolic content (g GAE/100 g DW)	0.28 ± 0.01 ^d^	0.36 ± 0.08 ^c^	0.54 ± 0.03 ^b^	0.71 ± 0.05 ^a^	0.07 ± 0.003 ^e^	0.16 ± 0.01 ^de^	0.06 ± 0.02 ^e^
Total flavonoid content (g CE/100 g DW)	0.08 ± 0.00 ^bc^	0.10 ± 0.01 ^b^	0.05 ± 0.01 ^c^	0.21 ± 0.03 ^a^	0.02 ± 0.00 ^d^	0.05 ± 0.01 ^c^	0.09 ± 0.01 ^bc^
DPPH, IC_50_ (μg/mL)	26.09 ± 0.33 ^ab^	21.9 ± 0.37 ^c^	19.33 ± 0.05 ^c^	15.7 ± 0.08 ^d^	36.2 ± 0.41 ^a^	30.13 ± 0.3 ^b^	38.0 ± 1.15 ^a^
Inhibition of linoleic acid peroxidation (%)	16.66 ± 1.56 ^bc^	18.44 ± 1.62 ^bc^	21.71 ± 3.12 ^b^	28.54 ± 2.31 ^a^	11.17 ± 0.71 ^d^	13.22 ± 0.73 ^c^	10.58 ± 0.6 ^d^
Reducing power for 10 mg/mL extract conc.	0.27 ± 0.06 ^b^	0.29 ± 0.06 ^b^	0.33 ± 0.06 ^a^	0.35 ± 0.07 ^a^	0.12 ± 0.04 ^c^	0.15 ± 0.05 ^cd^	0.09 ± 0.03 ^d^

Values are mean ± SD of three separate experiments. Different superscript letters within the same row indicate significant (*p* < 0.05) differences of means within the extracting solvents.

### 2.3. Total Flavonoids Content (TFC)

The TFC of bark, leaves and seeds extracts of *P. pinnata* with different solvents, ranged from 0.85–3.44, 0.10–0.61 and 0.02–0.21 g CE/100 g DW, respectively (Table 1), showing significant (*p *< 0.05) variations among different solvents. Of the extracts tested, aqueous methanol extract of bark (3.44 g CE/100g DW) showed the highest levels of total flavonoids, followed by leaves (0.61 g CE/100g DW) and seeds (0.21 g CE/100g DW). Variations of TFC among different solvent extracts might be attributed to the varied polarity of the solvents used, while difference of TFC among plant parts might be linked to the factors such as natural chemical composition of the material, maturity at harvest, soil state and post-harvest storage conditions [25]. The TFC of aqueous methanol extract of leaves (0.61 CE/100 g DW) in this study was higher than that reported previously for methanolic extract (0.24 g/100 g quercetin equivalent) of leaves of *P. pinnata* [23], however, no report was available on the TFC of bark or seed extracts of this plant for comparison with the data of present analysis.

### 2.4. HPLC Analysis of Phenolic Acids

Among the extraction solvents used, aqueous methanol was found to be the most efficient to extract higher amounts of total phenolics from bark, leaves and seeds of *P. pinnata*. Therefore, aqueous methanol extracts from the three tested parts were further analyzed by HPLC to quantify targeted individual phenolic acids (Table 2). Of the thirty phenolic compounds analyzed by HPLC, only seventeen were identified in bark, leaves and seeds extracts of *P. pinnata*. These compounds showed significant quantitative variations (*p* < 0.05) among different plant parts. Protocatechuic acid (2.43 mg/ 100 g DW), ferulic acid (2.17 mg/100 g DW), gallic acid (6.70 mg/100 g DW) and 4-hydroxy benzoic acid (2.15 mg/100 g DW) were the main phenolic acids in the bark. Others such as sorbic acid (1.21 mg/100 g DW), ferulic acid (1.12 mg/100 g DW), gallic acid (4.71 mg/100 g DW), salicylic acid (1.18 mg/100g DW) and, *p*-coumaric acid (1.19 mg/100g DW) were found to be the major phenolics in leaves, while vanillic acid (0.52 mg/100 g DW), gallic acid (0.65 mg/100 g DW), and tannic acid (0.57 mg/100 g DW) mainly existed in the seeds extract. 

The identified phenolic compounds are known to have antioxidant and medicinal properties [26,27,28]. Gallic acid, which is efficiently absorbed in human body, shows positive effects against cancer cells under *in vitro* conditions [29]. Another phenolic component, *p*-coumaric acid is believed to reduce the risk of stomach cancer by reducing the formation of carcinogenic nitrosamines [30]. There was no report available in literature on the composition of individual phenolic acids in the bark, leaves and seeds of *P. pinnata *for comparison of the results of our present experiment.

### 2.5. Percentage Inhibition of Linoleic Acid Peroxidation

The antioxidant activity of an extract can be assessed by its ability to retard linoleic acid peroxidation in a model system [31]. Therefore, this assay was used to assess the antioxidant activity of bark, leaves and seeds extracts of *P. pinnata*. Linoleic acid is a C-18 polyunsaturated fatty acid, under test conditions due to oxidation it produces peroxides which oxidize Fe^2+^ to Fe^3+^. The ferric ion (Fe^3+^) forms colored complex with SCN^−^, the intensity of which is examined colorimetrically by measuring the absorbance at 500 nm. A higher absorbance means a higher concentration of peroxides formed during the reaction, with subsequent sign of lower antioxidant activity.

**Table 2:** HPLC quantification of aqueous-methanol soluble phenolic components (mg/100 g DW) identified in different parts of *P. pinnata*.

Compounds	Bark	Leaves	Seeds
Protocatechuic acid	2.43 ± 0.14 ^a^	0.91 ± 0.06 ^b^	0.46 ± 0.16 ^c^
Sinapic acid	0.26 ± 0.01	ND	ND
Sorbic acid	0.34 ± 0.03 ^b^	1.21 ± 0.02 ^a^	0.02 ± 0.00 ^c^
Ellagic acid	1.50 ± 0.13 ^a^	0.10 ± 0.11 ^ab^	0.40 ± 0.16 ^b^
Ferulic acid	2.17 ± 0.16 ^a^	2.12 ± 0.13 ^a^	ND
Syringic acid	0.74 ± 0.05 ^a^	0.50 ± 0.06 ^b^	0.18 ± 0.02 ^c^
Vanillic acid	0.56 ± 0.06 ^a^	ND	0.52 ± 0.06 ^a^
Gallic acid	6.70 ± 0.31 ^a^	4.71 ± 0.24 ^b^	0.65 ± 0.06 ^c^
Chlorogenic acid	0.78 ± 0.04 ^a^	0.70 ± 0.05 ^a^	0.15 ± 0.16 ^b^
Gentisic acid	1.60 ± 0.04 ^a^	0.50 ± 0.16 ^b^	ND
Salicylic acid	0.14 ± 0.02 ^b^	1.18 ± 0.01 ^a^	ND
Caffeic acid	0.31 ± 0.06 ^a^	0.27 ± 0.02 ^a^	0.08 ± 0.01 ^b^
*p*-Coumaric acid	0.26 ± 0.02 ^b^	1.19 ± 0.05 ^a^	0.04 ± 0.16 ^c^
*m*-Coumaric acid	0.53 ± 0.08	ND	ND
Tannic acid	1.02 ± 0.06 ^a^	0.13 ± 0.03 ^c^	0.57 ± 0.03 ^b^
4-Hydroxybenzoic acid	2.15 ± 0.11 ^a^	0.29 ± 0.01 ^b^	0.11 ± 0.16 ^c^
4-Hydroxycinnamic acid	1.87 ± 0.06 ^a^	0.36 ± 0.02 ^b^	ND

Values are mean ± SD of three separate experiments. Different superscript letters within the same row indicate significant (*p* < 0.05) differences of means within the plant parts.

Table 1 summarizes the percent inhibition of linoleic acid peroxidation as exhibited by different solvent extracts of the tested parts of* P. pinnata*. Bark extracts exhibited higher inhibition of peroxidation ranging from 20.51 to 69.23%, followed by leaves (16.85–50.65%) and seeds (10.58–28.54%) extracts. The results were compared with butylated hydroxy toluene and ascorbic acid as positive controls, which offered inhibition of linoleic acid peroxidation at levels of 85.11% and 49.28%, respectively. Among the different solvent extracts tested, aqueous-methanol extract offered significantly (*p < *0.05) higher inhibition of peroxidation relative to the others. The efficacy of plant extracts obtained with different solvents for inhibition of linoleic acid peroxidation followed the order: aqueous methanol > absolute methanol > aqueous ethanol > absolute ethanol > aqueous acetone > absolute acetone > deionized water. 

### 2.6. DPPH Radical Scavenging Activity

DPPH radical scavenging assay is relatively a rapid and sensitive approach to evaluate the antioxidant activity of a specific compound or plant extract [32]. In this test, proton donor species such as phenolic antioxidants quench free radicals and the magnitude of which is measured colorimetrically in terms of IC_50_. The lower IC_50_ values reflect the greater potency for antioxidant activity of the extracts. The results for DPPH free radical scavenging activity (IC_50_ values) of* P. pinnata* bark, leaves and seeds extracts, produced by different solvents, are presented in Table 1. The bark extracts showed lower IC_50_ values (3.21–10.01 µg/mL) indicating higher radical scavenging activity, as compared to leaves (IC_50_ values 4.42–16.46 µg/mL) and seeds (IC_50_ values15.7–38.0 µg/mL) extracts. A stronger radical scavenging capacity of bark extracts, compared with leaves or seed extracts, might be linked to the presence of higher amounts of phenolic acids and flavonoids in this part of the plant. It is widely accepted that the, antioxidant activity of a plant material is strongly correlated with the amount of phenolics, as well as the degree of hydroxylation of the phenolics, and other chemicals structural features [33]. Among the extracts, aqueous-methanol extract was found to be superior and showed significantly (*p* < 0.05) stronger DPPH radical scavenging potential. The effectiveness of extracts obtained in different solvents in extraction of DPPH radical scavengers from the parts tested followed the order: aqueous methanol > absolute methanol > aqueous ethanol > absolute ethanol > aqueous acetone > absolute acetone > deionized water. Free radical scavenging capacity of leaves and bark extracts in the present study was found to be greater than that of the methanol extracts of leaves (IC_50_: 40 µg/mL) and stem (IC_50_: 250 µg/mL) of *P. pinnata* as investigated previously [21]. The free radical scavenging activity of leaves extract in this study was also found to be greater than that of methanolic extract of the leaves (IC_50_: 192 µg/mL) of *P. pinnata* reported in an earlier study [23].

### 2.7. Reducing Power of Extracts

The typical trends found during the measurement of reducing potential can describe some aspects of antioxidant activity of the plant extracts. In this method, ferric (Fe^3+^) ions are reduced to ferrous (Fe^2+^) ions which result change in color from yellow to bluish green. The intensity of color depends on the reducing potential of the compounds present in the extract medium. Greater the intensity of the color, greater will be the absorption; consequently, greater will be the antioxidant activity [34]. 

The reducing potential of different solvent extracts (concentrations varying from 2.5–10.0 mg/mL) from selected parts of *P. pinnata* gradually increased showing a concentration-dependent effect (data not shown). The reducing powers recorded for different solvent extracts (at concentration 10 mg/mL) from bark, leaves and seeds of the subject plant are presented in Table 1. As expected, the aqueous-methanol extract of bark (absorbance value = 1.73) exhibited highest reducing power followed by leaves (absorbance value = 1.64) and seeds (absorbance value = 0.35) extracts at the same concentration. The variation in reducing powers among different solvent extracts was found to be statistically significant (*p* < 0.05). The reducing power of aqueous methanolic bark extract in this study was comparable to that observed by Babu *et al*. [23], for flower extracts of *P. pinnata*, however, there are no earlier reports available on the reducing potential of leaves and seeds of this plant with which to compare the present results.

### 2.8. Antimicrobial Activity

Antimicrobial activity of the bark, leaves, and seeds extracts of *P. pinnata *against six pathogenic bacteria and fungi is shown in Table 3, Table 4 and Table 5, respectively. The antimicrobial activity of various solvent extracts for selected plant part varied significantly (*p* < 0.05). Among the different solvent extracts, the aqueous methanol extract of bark exhibited the strongest antimicrobial activity followed by leaves and seeds extracts with zone of inhibition ranging from 16.7 to 26.2 mm, 10.2 to 16.2 mm and 8.8 to 11.5 mm, respectively. The MIC values for aqueous methanol extracts from bark, leaves and seeds ranged from 22 to 36 mg/mL, 72 to 90 mg/mL and 93 to 109 mg/mL, respectively. The antimicrobial activity in terms of zone of inhibition and MIC data of aqueous methanol extract of bark was comparable with the respective standard drugs amoxicillin and flumequine. The superior antimicrobial activity of aqueous-methanol extract of *P. pinnata* bark might be partly due to the higher contents of phenolic acids and flavonoids in this extract. Flavonoids are known to retard the growth of microorganism through inhibiting their nucleic acid synthesis, cytoplasmic membrane function and energy metabolism [35,36]. Different plant parts exhibited antimicrobial activity but to varying extent**. **These differences can be attributed to the accumulation of different antimicrobial agents in different parts of *P. pinnata.* Some earlier reports showed that the changes in chemical composition of an extract directly affect its biological activities [37]. In our study the antimicrobial activity of *P.pinnata* leaves extract was found to be stronger than reported in an earlier study on this plant [38].

## 3. Experimental

### 3.1. Sample Collection and Preparation of Extracts

Bark, leaves and seeds samples of* P. pinnata* were collected from the fully mature plants grown in the vicinity of the University of Agriculture Faisalabad, Pakistan. The specimens were further identified and authenticated by the Department of Botany, University of Agriculture Faisalabad, Pakistan. Air-dried samples of bark, leaves and, seeds, were ground to a fine powder (80 mesh) in a grinding mill (Tector-Cemotec 1090 sample mill, Hognas, Sweden). For each of the dried parts (bark, leaves and seeds), material (20 g) was separately extracted with 200 mL of seven different solvents [absolute methanol, absolute ethanol, absolute acetone, aqueous methanol (methanol-water, 80:20, v/v), aqueous ethanol (ethanol-water, 80:20, v/v), aqueous acetone (acetone-water, 80:20, v/v) and deionized water] using an orbital shaker (Gallenkamp, Surrey, UK) for 8 h at room temperature. The extracts were separated from the solids by filtration with Whatman No. 1 filter paper. The remaining solids were extracted twice with the same solvent and extracts combined. The extracts were concentrated under reduced pressure at 45 °C, in a rotary evaporator (EYELA, Tokyo, Japan). Concentrated extracts were stored in a refrigerator at 4 °C) until analyzed.

### 3.2. Determination of Total Phenolic Content (TPC)

The TPC in the extracts was assessed using Folin-Ciocalteu reagent procedure as described by Chaovanalikit and Wrolstad [39]. 

### 3.3. Determination of Total Flavonoid Content (TFC)

The TFC in the extracts was determined following the procedure of Dewanto *et al.* [40].

### 3.4. High Performance Liquid Chromatography (HPLC) Analysis

Analysis of phenolic acids in the plant extracts obtained with aqueous-methanol was performed on Varian HPLC using ODS2 C18 reversed phase column (250 × 4.6 mm) [41]. HPLC assay was conducted using acidified acetonitrile (99.5%) as mobile phase with constant flow rate of 1 mL/min in isocratic mode. Sample injection volume was 20 µL. The detection was performed at 280 nm. Phenolic compounds of each sample were identified by comparing their relative retention times with those of the standard mixture chromatogram. The concentration of an individual compound was calculated on the basis of peak area measurement and then converted to mg phenolics/100 g DW.

**Table 3:** Antimicrobial activity of different solvent extracts from bark of *P. pinnata*.

Tested organisms	Bark extracts								
	Absoluteethanol	Aqueous ethanol	Absolutemethanol	Aqueous methanol	Absoluteacetone	Aqueous acetone	Deionizedwater	Amoxicillin	Flumequine
	**Diameter of inhibition zone (mm)**								
*Pseudomonas stutzeri*	12.8 ± 0.8 ^bc^	16.3 ± 0.7 ^b^	15.6 ± 0.5 ^b^	21.5 ± 0.5 ^a^	11.6 ± 0.5 ^c^	12.3 ± 0.8 ^bc^	11.0 ± 0.3 ^c^	24.2± 1.2 ^a^	-----
*Pseudomonas aeruginosa*	10.3 ± 0.4 ^bc^	13.9 ± 0.4 ^b^	12.6 ± 0.4 ^bc^	19.3 ± 0.4 ^a^	9.2 ± 0.3 ^c^	9.8 ± 0.3 ^c^	10.0 ± 0.4 ^c^	21.3± 0.9 ^a^	-----
*Escherichia coli*	8.8 ± 0.4 ^c^	11.3 ± 0.3 ^b^	10.8 ± 0.4 ^b^	16.7 ± 0.5 ^a^	8.1 ± 0.3 ^c^	8.4 ± 0.3 ^c^	8.0 ± 0.3 ^c^	18.2± 1.0 ^a^	-----
*Aspergillus orazae*	13.1 ± 0.2 ^bc^	17.5 ± 0.2 ^b^	16.5 ± 0.7 ^b^	26.2 ± 0.8 ^a^	11.8 ± 0.4 ^bc^	12.1 ± 0.5 ^bc^	9.8 ± 0.3 ^c^	-----	28.5 ± 1.2 ^a^
*Aspergillus niger*	12.9 ± 0.8 ^bc^	14.1 ± 0.6 ^ab^	13.5 ± 0.5 ^b^	24.7 ± 0.8 ^ab^	10.9 ± 0.4 ^bc^	9.0 ± 0.3 ^c^	8.5 ± 0.2 ^c^	-----	26.2 ± 1.2 ^a^
*Fusarium solani*	12.0 ± 0.7 ^b^	13.1 ± 0.5 ^b^	11.7 ± 0.4 ^b^	22.9 ± 0.5 ^a^	9.2 ± 0.5 ^bc^	8.1 ± 0.3 ^c^	7.5 ± 0.2 ^c^	-----	24.3 ± 1.2 ^a^
	**Minimum inhibitory concentration (mg/mL)**								
*Pseudomonas stutzeri*	51 ± 2 ^b^	48 ± 2 ^b^	36 ± 2 ^bc^	26 ± 1 ^c^	62 ± 1 ^a^	58 ± 2 ^a^	60 ± 2 ^a^	23 ± 1 ^d^	-----
*Pseudomonas aeruginosa*	60 ± 3 ^c^	58 ± 3 ^c^	39 ± 1 ^cd^	29 ± 3 ^d^	80 ± 2 ^a^	71 ± 1 ^b^	76 ± 3 ^ab^	25 ± 1 ^d^	-----
*Escherichia coli*	66 ± 3 ^ab^	65 ± 1 ^ab^	41 ± 2 ^c^	36 ± 2 ^c^	86 ± 2 ^a^	78 ± 2 ^ab^	79 ± 2 ^b^	32 ± 2 ^d^	-----
*Aspergillus orazae*	48 ± 1 ^ab^	46 ± 2 ^ab^	42 ± 2 ^b^	22 ± 1 ^b^	24 ± 1 ^a^	52 ± 2 ^a^	54 ± 1 ^a^	-----	20 ± 1 ^c^
*Aspergillus niger*	51 ± 3 ^ab^	48 ± 3 ^ab^	46 ± 1 ^ab^	29 ± 2 ^b^	28 ± 2 ^a^	65 ± 3 ^a^	67 ± 2 ^a^	-----	28 ± 1 ^c^
*Fusarium solani*	58 ± 2 ^ab^	55 ± 2 ^ab^	53 ± 3 ^ab^	36 ± 3 ^b^	36 ± 2 ^a^	72 ± 3 ^a^	74 ± 3 ^a^	-----	33 ± 2 ^c^

Values are mean ± SD of three separate experiments. Different superscript letters within the same row indicate significant (*p* < 0.05) differences of means within the extracting solvents.

**Table 4:** Antimicrobial activity of different solvent extracts from leaves of *P. pinnata*.

Tested organisms	Leaves extracts								
	Absolute ethanol	Aqueous ethanol	Absolute methanol	Aqueous methanol	Absolute acetone	Aqueous acetone	Deionized water	Amoxicillin	Flumequine
	**Diameter of inhibition zone (mm)**								
*Pseudomonas stutzeri*	10.6 ± 0.3 ^c^	12.1 ± 0.6 ^bc^	12.6 ± 0.4 ^bc^	16.2 ± 0.8 ^b^	10.0 ± 0.4 ^c^	11.0 ± 0.5 ^c^	9.20 ± 0.2 ^c^	24.2 ± 1.2 ^a^	-----
*Pseudomonas aeruginosa*	9.3 ± 0.2 ^bc^	10.6 ± 0.4 ^bc^	10.1 ± 0.3 ^bc^	14.5 ± 0.5 ^b^	8.20 ± 0.3 ^c^	9.30 ± 0.4 ^bc^	7.50 ± 0.2 ^c^	21.3 ± 0.9 ^a^	-----
*Escherichia coli*	8.0 ± 0.1 ^bc^	8.40 ± 0.3 ^bc^	8.20 ± 0.3 ^bc^	10.2 ± 0.4 ^b^	7.50 ± 0.2 ^bc^	8.50 ± 0.3 ^bc^	6.90 ± 0.3 ^c^	18.2 ± 1.0 ^a^	-----
*Aspergillus orazae*	12.6 ± 0.4 ^bc^	14.0 ± 0.8 ^b^	12.3 ± 0.7 ^bc^	15.7 ± 0.8 ^b^	10.9 ± 0.5 ^bc^	11.6 ± 0.5 ^bc^	9.50 ± 1.2 ^c^	-----	28.5 ± 1.2 ^a^
*Aspergillus niger*	11.0 ± 0.2 ^bc^	10.8 ± 0.5 ^bc^	10.6 ± 0.4 ^bc^	13.5 ± 0.7 ^b^	9.00 ± 0.4 ^bc^	8.60 ± 0.4 ^bc^	8.30 ± 0.3 ^c^	-----	26.2 ± 1.2 ^a^
*Fusarium solani*	9.4 ± 0.1 ^bc^	10.0 ± 0.4 ^bc^	7.00 ± 0.2 ^c^	11.6 ± 0.4 ^b^	8.00 ± 0.3 ^bc^	7.80 ± 0.2 ^bc^	7.30 ± 0.2 ^c^	-----	24.3 ± 1.1 ^a^
	**Minimum inhibitory concentration (mg/mL)**								
*Pseudomonas stutzeri*	92 ± 1 ^a^	89 ± 2 ^b^	86 ± 3 ^b^	80 ± 1 ^b^	106 ± 1 ^a^	98 ± 1 ^a^	109 ± 1 ^a^	23 ± 1 ^c^	-----
*Pseudomonas aeruginosa*	96 ± 2 ^a^	92 ± 3 ^b^	96 ± 2 ^b^	88 ± 3 ^b^	110 ± 1 ^a^	108 ± 1 ^a^	119 ± 2 ^a^	25 ± 1 ^c^	-----
*Escherichia coli*	127 ± 2 ^a^	122 ± 1 ^b^	121 ± 1 ^b^	90 ± 2 ^b^	127 ± 3 ^a^	113 ± 2 ^a^	129 ± 1 ^a^	32 ± 2 ^c^	-----
*Aspergillus orazae*	90 ± 2 ^ab^	85 ± 2 ^ab^	81 ± 2 ^ab^	72 ± 3 ^b^	110 ± 1 ^a^	108 ± 1 ^a^	109 ± 3 ^a^	-----	20 ± 1 ^c^
*Aspergillus niger*	95 ± 2 ^ab^	90 ± 2 ^ab^	90 ± 2 ^ab^	81 ± 2 ^b^	112 ± 2 ^a^	110 ± 1 ^a^	108 ± 1 ^a^	-----	28 ± 1 ^c^
*Fusarium solani*	112 ± 2 ^ab^	109 ± 2 ^ab^	106 ± 1 ^ab^	90 ± 2 ^b^	119 ± 1 ^a^	116 ± 1 ^a^	117 ± 2 ^a^	-----	33 ± 2 ^c^

Values are mean ± SD of three separate experiments. Different superscript letters within the same row indicate significant (*p* < 0.05) differences of means within the extracting solvents.

**Table 5:** Antimicrobial activity of different solvent extracts from seeds of *P. pinnata*.

Tested organisms	Seeds extracts								
	Absolute Ethanol	Aqueous ethanol	Absolute methanol	Aqueous methanol	Absolute acetone	Aqueous acetone	Deionized water	Amoxicillin	Flumequine
	**Diameter of inhibition zone (mm)**								
*Pseudomonas stutzeri*	9.3 ± 0.3 ^bc^	10.3 ± 0.4 ^bc^	9.0 ± 0.41 ^bc^	11.3 ± 0.3 ^b^	7.5 ± 0.3 ^bc^	7.8 ± 0.3 ^bc^	7.0 ± 0.2 ^c^	24.2 ± 1.2 ^a^	-----
*Pseudomonas aeruginosa*	9.0 ± 0.2 ^bc^	9.8 ± 0.3 ^bc^	8.0 ± 0.3 ^bc^	10.0 ± 0.2 ^b^	7.0 ± 0.3 ^bc^	7.2 ± 0.3 ^bc^	6.5 ± 0.2 ^c^	21.3 ± 0.9 ^a^	-----
*Escherichia coli*	8.4 ± 0.2 ^bc^	9.0 ± 0.3 ^b^	7.9 ± 0.4 ^bc^	8.8 ± 0.3 ^b^	6.5 ± 0.3 ^bc^	7.0 ± 0.2 ^bc^	6.2 ± 0.1 ^c^	18.2 ± 1.0 ^a^	-----
*Aspergillus orazae*	10.6 ± 0.3 ^b^	11.1 ± 0.4 ^b^	10.1 ± 0.4 ^ab^	11.5 ± 0.3 ^b^	9.0 ± 0.4 ^b^	9.5 ± 0.3 ^bc^	8.2 ± 0.3 ^c^	-----	28.5 ± 1.2 ^a^
*Aspergillus niger*	10.2 ± 0.4 ^b^	10.8 ± 0.4 ^b^	10.0 ± 0.5 ^b^	11.2 ± 0.3 ^b^	7.9 ± 0.4 ^c^	7.5 ± 0.3 ^c^	7.1 ± 0.2 ^c^	-----	26.2 ± 1.2 ^a^
*Fusarium solani*	9.5 ± 0.5 ^bc^	9.9 ± 0.3 ^b^	9.1 ± 0.5 ^bc^	10.1 ± 0.4 ^b^	6.8 ± 0.3 ^c^	7.2 ± 0.2 ^cc^	6.4 ± 0.1 ^c^	-----	24.3 ± 1.1 ^a^
	**Minimum inhibitory concentration (mg/mL)**								
*Pseudomonas stutzeri*	109 ± 20 ^ab^	105 ± 4 ^b^	112 ± 2 ^ab^	106 ± 2 ^b^	135 ± 2 ^a^	119 ± 2 ^ab^	125 ± 2 ^ab^	23 ± 1 ^c^	-----
*Pseudomonas aeruginosa*	104 ± 3 ^c^	102 ± 4 ^a^	120 ± 4 ^b^	109 ± 4 ^c^	140 ± 3 ^a^	122 ± 4 ^b^	130 ± 2 ^ab^	25 ± 1 ^c^	-----
*Escherichia coli*	118 ± 2 ^ab^	101 ± 3 ^b^	126 ± 2 ^ab^	112 ± 3 ^ab^	146 ± 2 ^a^	127 ± 4 ^ab^	141 ± 4 ^a^	32 ± 2 ^c^	-----
*Aspergillus orazae*	95 ± 3 ^b^	96 ± 3 ^b^	101 ± 4 ^ab^	93 ± 2 ^b^	112 ± 4 ^a^	108 ± 2 ^ab^	114 ± 2 ^a^	-----	20 ± 1 ^c^
*Aspergillus niger*	97 ± 2 ^b^	98 ± 4 ^b^	105 ± 2 ^a^	98 ± 1 ^b^	117 ± 3 ^a^	110 ± 3 ^ab^	117 ± 2 ^a^	-----	28 ± 1 ^c^
*Fusarium solani*	99 ± 3 ^b^	102 ± 3 ^b^	107 ± 3 ^a^	101 ± 2 ^b^	118 ± 2 ^ab^	113 ± 2 ^ab^	132 ± 2 ^a^	-----	33 ± 2 ^c^

Values are mean ± SD of three separate experiments. Different superscript letters within the same row indicate significant (*p* < 0.05) differences of means within the extracting solvents.

### 3.5. Antioxidant Activity Determination in Linoleic Acid System

The antioxidant activity of plant extracts was determined in terms of measurement of % inhibition of peroxidation in linoleic acid system following a method of Osawa and Namiki [42]. 

### 3.6. Determination of Reducing Power

The reducing power of the extracts was determined according to the procedure described by Yen *et al*. [31].

### 3.7. DPPH Radical Scavenging Assay

The radical scavenging activity of the plant extracts against 2,2-diphenyl-1-picrylhydrazyl radical was measured by a slightly modified method as previously described by Ayoola *et al.* [43]. Aliquots of various concentrations (10–100 µg/mL) of the extracts were prepared in methanol. Extract (1 mL) was placed in a test tube, and methanol (3 mL) was added, followed by 1 mM DPPH in methanol (0.5 mL). A blank solution was prepared containing the same amount of methanol and DPPH. After a 30 min incubation period at room temperature the absorbance was read against blank at 517 nm using a spectrophotometer. Inhibition of free radical by DPPH in percent (%) was calculated using following formula:

            % inhibition of DPPH^•^ = {[Ab − Aa]/Ab} × 100

where Ab is the absorption of the blank sample and Aa is the absorption of the extract. IC_50_ values, which represented the extract concentration providing 50% inhibition of DPPH radicals, were calculated from the plot of inhibition percentage against extract concentration.

### 3.8. Antimicrobial Activity

The plant extracts were individually tested against a set of common pathogenic microorganisms, including three Gram-negative bacteria: *Pseudomonas aeruginosa*,* Pseudomonas stutzeri*, *Escherichia coli* and three fungi: *Aspergillus orazae*, *Aspergillus niger*, *Fusarium solani*. The pure bacterial and fungal strains were obtained from National Institute for Biotechnology and Genetic Engineering (NIBGE), Faisalabad, Pakistan. Bacterial strains were cultured overnight at 37 °C in Nutrient agar (NA, Oxoid, Basingstoke, UK) while fungal strains were cultured overnight at 30 °C using Potato dextrose agar (PDA, Oxoid). Antimicrobial activity of the extracts was evaluated using disc diffusion method and micro dilution broth method.

### 3.9. Disc Diffusion Method

The antimicrobial activities of the bark, leaves and seeds of *P. pinnata* were determined by agar disc diffusion method [44]. Briefly, 100 µL of suspension of tested microorganisms, containing 10^8^ colony-forming units (cfu/mL of bacteria cells and 10^4^ cfu/mL spores of fungal strains spread on nutrient agar (NA) and potato dextrose agar (PDA) medium, respectively. The sterilized filter paper discs (6 mm in diameter) were impregnated with 20 µL of 100 mg/mL extract (2 mg/disc), were arranged on the surface of the agar plates which had previously been inoculated with the tested microorganisms. Disc without samples were used as a negative control. Amoxycillin (30 µg/disc) and flumequine (30 µg/disc) were used as positive references for bacteria and fungi, respectively. The plates were incubated at 37 °C for 24 h for bacteria and at 30 °C for 48 h for fungal strains. Antimicrobial activity was evaluated by measuring the diameter of the growth inhibition zones in millimeters (including disc diameter of 6 mm) for the test organisms and comparing to the controls. The measurement of inhibition zones was carried out using three sample replications, and values presented are the average of three replicates.

### 3.10. Determination of Minimum Inhibitory Concentration

For the determination of MIC, which represents the minimum concentration that completely inhibits the growth of microorganisms; a micro-dilution broth susceptibility assay was used [45]. All tests were performed in Nutrient broth (NB) and Sabouraud dextrose broth (SDB) supplemented with Tween 80 detergent to a final concentration of 0.5% (v/v) for bacteria and fungi, respectively. Bacterial strains were cultured overnight at 37 °C in NB and the fungi were cultured overnight at 30 °C in SDB. Dilutions series were prepared from 5 to 100 mg/mL of the extracts. Each concentration of extract (0.1 mL) was added to NB and SDB (9 mL of each) for bacteria and fungi, respectively, containing standardized bacterial or fungal cell test organisms (0.1 mL). The tubes were incubated at 37 °C for 24 h for bacteria, and at 30 °C for 48 h for fungi. Positive controls were equally set up by using solvents and test organisms without extracts. The same test was performed simultaneously for the growth control (NB + Tween 80) and sterility control (NB + Tween 80 + test extract). Amoxycillin was used as a reference compound for antibacterial and flumequine for antifungal activities. The tube with least concentration of extract without growth after incubation was taken and recorded as the minimum inhibitory concentrations. 

### 3.11. Statistical Analysis

Data were analyzed using one-way analysis of variance ANOVA using Minitab 2000 Version 13.2 statistical software (Minitab Inc., State College, PA, USA) at 5% significance level.

## 4. Conclusions

The current study was the first attempt revealing the variations of biological activities among bark, leaves and seeds of *P. pinnata* using a range of extraction solvents. Aqueous methanol was established to be the most effective solvent to recover higher amounts of phenolics from different parts of *P. pinnata* compared with other solvents. Besides, it was concluded that extracts from bark of this plant had higher antioxidant and antimicrobial activities and concentration of phenolic acids among others, regardless of the extraction solvent employed. In light of the present results *P. pinnata* can be used to isolate high-value bio-actives that may serve as leads for the development of new antimicrobial drugs and functional foods for pharmaceutical and nutraceutical uses. Further detailed studies on the isolation and therapeutic properties of bioactives of this plant using some *in vivo *models is recommended to establish specific applications and to formulate new and potent antimicrobial drugs of natural origin.

## References

[1] Liu Q., Yao H. (2007). Antioxidant activities of barley seeds extracts. Food Chem..

[2] Iqbal S., Bhanger M.I., Anwar F. (2007). Antioxidant properties and components of bran extracts from selected wheat varieties commercially available in Pakistan. LWT-Food Sci. Technol..

[3] Chopra R.N., Nayer S.L., Chopra I.C. (1992). Glossary of Indian Medicinal Plants.

[4] Bruneton J. (1995). Pharmacognosy, Phytochemistry, Medicinal Plants.

[5] Pathak V.P., Saini T.R., Khanna R.N. (1983). Isopongachromene, a chromenoflavone from *Pongamia glabra *seeds. Phytochemistry.

[6] Parekh J., Nair R., Chanda S. (2005). Preliminary screening of some folkloric plants from Western India for potential antimicrobial activity. Indian J. Pharmacol..

[7] Parmar B.S., Sahravat K.L., Mukarjee S.K. (1976). *Pongamia glabra*: Constituents and uses. J. Sci. Ind. Res..

[8] Punitha R., Manoharan S. (2006). Antihyperglycemic and antilipidperoxidative effects of *Pongamia pinnata* (Linn.) Pierre flowers in alloxan induced diabetic rats. J. Ethnopharmacol..

[9] Ballal M. Screening of medicinal plants used in rural folk medicine for treatment of diarrhea. http://www.pharmoinfo.net.

[10] Tanaka T., Linuma M., Yuki K., Fujy Y., Mizuno M. (1992). Flavonoids in root bark of *Pongamia pinnata*. Phytochemistry.

[11] Carcache B.E.J., Kang Y.H., Park E.J., Su B.N., Kardono L.B.S., Riswan S., Fong H.H.S., Pezzuto J.M., Kinghorn A.D. (2003). Constituents of the stem bark of *Pongamia pinnata* with the potential to induce quinine reductase. J. Nat. Prod..

[12] Rastogi R.P., Malhotra B.N. (2001). Compendium an Medicinal Plants.

[13] Chauhan D., Chauhan J.S. (2002). Flavonoid glycosides from *Pongamia pinnata*. Pharm. Biol..

[14] Li L., Li X., Shi C., Deng Z., Fu H., Proksh P., Lin W. (2006). Pongamones A–E, five flavonoids from the stems of a mangrove plant *Pongamia pinnata*. Phytochemistry.

[15] Tanaka T., Linuma M., Yuki K., Fujy Y., Mizuno M. (1991). Two new β-hydroxychalcones from the root bark of *Pongamia pinnata*. Chem. Pharm. Bull..

[16] Kitagawa I., Zang R., Hori K., Tsuchiya K., Shibuya H. (1992). Indonesian medicinal plants. II. Chemical structures of pongapinones A and B, two new phenylpropanoids from the bark of *Pongamia pinnata* (Papilionaceae). Chem. Pharm. Bull..

[17] Yadav P.P., Ahmed G., Mourya R. (2004). Furanoflavonoids from *Pongamia pinnata* fruits. Phytochemistry.

[18] Shabir G., Anwar F., Sultana B., Khalid Z.M., Afzal M., Khan Q.M., Ashrafuzzaman M. (2011). Antioxidant and antimicrobial attributes and phenolics of different solvent extracts from leaves, flowers and bark of Gold Mohar (*Delonix regia*). Molecules.

[19] Ahmad N., Anwar F., Hameed S., Boyce M.C. (2011). Antioxidant and antimicrobial attributes of different solvent extracts from leaves and flowers of akk[calotropis procera (Ait.) Ait.F.)]. J. Med. Plants Res..

[20] Siddhuraju P., Becker K. (2003). Antioxidant properties of various extracts of total phenolic constituents from three different agroclimatic origins of drumstick tree (*Moringa oleifera* Lam.) leaves. J. Agric. Food Chem..

[21] Sagwan S., Rao D.V., Sharma R.A. (2011). *In-vitro* and *In-vivo* antioxidant activity and total phenolic content of * Pongamia pinnata* (L.) Pierre: An important medicinal plant. Int. J. Biotechnol..

[22] Babu D.R., Rao G.N. (2010). *In vitro* studies on extracts of *Pongamia pinnata* (L) Pierre flowers as a potent antioxidant. Int. J. Agric. Food Sci. Technol..

[23] Gupta V., Sharma M. (2011). Screening of three Indian medicinal plant extracts for antioxidant activity. Int. J. Inst. Pharm. Life Sci..

[24] Jaffery E.H., Brown A.F., Kurilich A.C., Keek A.S., Matusheski N., Klein B.P. (2003). Variation in content of bioactive components in broccoli. J. Food Compos. Anal..

[25] Huang D., Ou B., Prior R.L. (2005). The chemistry behind antioxidant capacity assays. J. Agric. Food Chem..

[26] Rawat S., Jugran A., Giri L., Bhatt I.D., Rawal R.S. (2011). Assessment of antioxidant properties in fruits of *Myrica esculenta*: A popular wild edible species in Indian Himalayan region. Evid. Based Complement. Alternat. Med..

[27] Zheng W., Wang S.Y. (2001). Antioxidant activity and phenolic compounds in selected herbs. J. Agric. Food Chem..

[28] Merkl R., Hrádková I., Filip V., Šmidrkal J. (2010). Antimicrobial and antioxidant properties of phenolic acids alkyl esters. Czech J. Food Sci..

[29] Tomas-Barberan F., Clifford M.N. (2000). Dietary hydroxybenzoic acid derivatives—Nature, occurrence and dietary burden. J. Sci. Food Agric..

[30] Ferguson L.R., Shuo-Tun Z., Harris P.J. (2005). Antioxidant and antigenotoxic effects of plant cell wall hydroxycinnamic acids in cultured HT-29. Mol. Nutr. Food Res..

[31] Yen G.C., Duh P.D., Chuang D.Y. (2000). Antioxidant activity of anthraquinones and anthrone. Food Chem..

[32] Qureshi M.N., Kuchekar B.S., Logade N.A., Haleem M.A. (2010). *In-vitro* antioxidant and *in-vivo* epatoprotective activity of *Leucas ciliata* leaves. Rec. Nat. Prod..

[33] Demiray S., Pintado M.E., Castro P.M.L. (2009). Evaluation of phenolic profiles and antioxidant activities of Turkish medicinal plants: *Tiliaargentea*, *Crataegi folium* leaves and *Polygonum bistorta* roots. World Acad. Sci. Eng. Technol..

[34] Zou Y., Lu Y., Wei D. (2004). Antioxidant activity of a flavonoid rich extract of *Hypericum perforatum* L.* in vitro*. J. Agric. Food Chem..

[35] Thiem B., Grosslinka O. (2000). Antimicrobial activity of *Rubus chamaemorus* leaves. Fitoterapia.

[36] Tim T.P., Lamb A.J. (2005). Antimicrobial activity of flavonoids. Int. J. Antimicrob. Agents.

[37] Celiktasa Y.O., Nartop P., Gurel A., Bedir E., Vardar-Sukan F. (2007). Determination of phenolic content and antioxidant activity of extracts obtained from *Rosmarinus officinalis*’ calli. J. Plant Physiol..

[38] Chandrashekar K.S., Prasanna K.S. (2010). Antimicrobial activity of *Pongamia pinnata* leaves. Int. J. Med. Res..

[39] Chaovanalikit A., Wrolstad R.E. (2004). Total anthocyanins and total phenolics of fresh and processed cherries and their antioxidant properties. J. Food Sci..

[40] Dewanto V., Wu X., Adom K.K., Liu R.H. (2002). Thermal processing enhances the nutritional value of tomatoes by increasing total antioxidant activity. J. Agric. Food Chem..

[41] Chapuis-Lardy L., Contour-Ansel D., Bernhard-Reversat F. (2002). High-performance liquid chromatography of water-soluble phenolics in leaf litter of three *Eucalyptus hybrids* (Congo). Plant Sci..

[42] Osawa T., Namiki M. (1981). A novel type of antioxidant isolated from leaf wax of eucalyptus leaves. Agric. Biol. Chem..

[43] Ayoola G.A., Sofidiya T., Odukoya O., Coker H.A.B. (2006). Phytochemical screening and free radical scavenging activity of some Nigerian medicinal plants. J. Pharm. Sci. Pharm. Pract..

[44] NCCLS (National Committee for Clinical Laboratory Standards) (1997). Performance Standards for Antimicrobial Disc Susceptibility Test.

[45] NCCLS (National Committee for Clinical Laboratory Standards) (1999). Performance Standards for Antimicrobial Susceptibility Test.

